# Investigating the anti-streptococcal biofilm effect of ssDNA aptamer-silver nanoparticles complex on a titanium-based substrate†

**DOI:** 10.1039/d2ra04112j

**Published:** 2022-09-15

**Authors:** Barumand Hosseini, Mandana Behbahani, Ghasem Dini, Hassan Mohabatkar, Mehrnaz Keyhanfar

**Affiliations:** Department of Biotechnology, Faculty of Biological Science and Technology, University of Isfahan Isfahan 81746-73441 Iran ma.behbahani@ast.ui.ac.ir +98-31-37932342 +98-31-37934327; Department of Nanotechnology, Faculty of Chemistry, University of Isfahan Isfahan 81746-73441 Iran

## Abstract

*Streptococcus mutans* is a commensal and opportunistic pathogen that causes several diseases by forming a biofilm in humans and animals in many areas such as nasopharyngeal, cardiac valves, lungs, and oral cavity. Biofilms are very important in prosthetic infections associated with medical implants. The use of nanoparticles is one of the evolving fields in biofilm targeting. Silver nanoparticles can be used for biofilm targeting due to their inherent antimicrobial properties. Hybridization of nanoparticles with small molecules increases their biological properties and makes them multifunctional. The present investigation aimed to design an appropriate silver nanoparticles–aptamer complex that binds to the surface receptors of streptococcal strains. For this reason, silver nanoparticles with particle sizes in a range of 50 to 70 nm were synthesized and connected to a designed aptamer with a streptavidin–biotin linker. Then, the effect of the complex was investigated on the *S. mutans* biofilm formed on the surface of a medical-grade titanium substrate. The silver nanoparticles–aptamer complex at a concentration of 100 μg mL^−1^ after 48 h inhibited 43% of the biofilm formation and degraded 63% of the formed biofilm. Also, the cell availability reached 96% and the complex was stable in cell medium culture for 360 min. It was concluded that this complex could be a good candidate for removing the formed biofilms on the surface of titanium implants.

## Abstract 2

Silver nanoparticles attached to the specific aptamer for*Streptococcus mutans,* can significantly remove the biofilm of this bacterium attached to the surface of the titanium implant.

## Graphical abstract

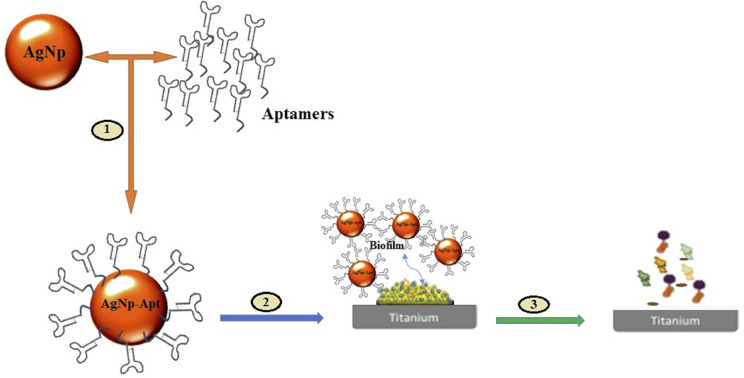

## 1 Introduction

Members of *Streptococcus* are Gram-positive, commensal, and opportunistic pathogens that cause various infections in humans and animals.^1^ Biofilm growth allows bacteria to be resistant to the host immune system and antibiotics, which means that eradication and clinical treatment of these infections are difficult.^2^ Infections associated with medical implants are prevalent and have medical complexity. Treatment of these infections is risky and their recurrence is common,^3^ because microorganisms attach to the surface of medical implants and form a biofilm that exhibits specific growth rates and morphology compared to individual or planktonic states.^4^

In humans, according to the National Institutes of Health, biofilms account for more than 80% of all microbial infections.^5^ The main medical challenge in the treatment of biofilms is that the destruction of formed biofilms is very difficult due to their high resistance to antibiotics.^6^ Compared to single bacteria, the bacteria present in a seven-day biofilm are 500 to 5000 times less sensitive to antimicrobial agents.^7^ There is a great deal of controversy and uncertainty in the diagnosis, prevention, and treatment of infections associated with implant biofilms. When a biofilm is formed, it is costly to remove and destroy, because the length of time a patient is hospitalized increases, long-term use of antibiotics occurs, and eventually, surgery is performed in some cases.^8^

In the United States, microbial biofilms cause 1.7 million nosocomial infections per year, costing approximately $11 billion.^9^ Conventional biomedical devices and implants have disadvantages such as short lifetime, large equipment size, and potential safety hazards.^10^ The application of nanotechnology and nanosystems in healthcare and biomedical devices improves the quality of this field.^11^ Biofilm formation and its growth on implants cause inefficiency of their proper actions and lead to diseases that can be fatal.^12^ Medical grade titanium and its alloys are used to make implants such as heart valves.^13^ Endocarditis is caused by the growth of bacteria on the inner surface of the heart valve. Biofilm creation on the heart prosthetic valve led to prosthetic valve endocarditis.^14^ This is a big risk for replacement heart valves since it is associated with a high mortality rate. The prevalence of this disease varies based on the study population and the age of the population. Among people with artificial valves, the risk is 1% in the first 12 months after surgery and 3% in the 60 months after surgery.^15^

Prosthetic valves are likely to develop infectious endocarditis in the first two months after surgery and up to 12 months after it.^16^ In general, *streptococci* and *staphylococci* are common causes of endocarditis and account for 80% of all cases. Among them, *viridans streptococci* cause 12.1% of all cases of heart prosthetic valve endocarditis.^16,17^ A group of *viridans streptococci* and *Streptococcus bovis* cause uncomplicated prosthetic endocarditis.^18^

Advances in the biological identification of biofilms have led to the development of new therapeutic strategies for biofilm targeting.^19^ The use of exopolysaccharide-degrading enzymes is a strategy that has increased the effectiveness of antimicrobials.^20^ Another anti-biofilm method is the use of hydrolyzer-bacteriophage-encoding peptidoglycan hydrolases.^21^ The use of nanoparticles is one of the new and evolving fields in biofilm targeting. Nanoparticles are in the spotlight due to their inherent antimicrobial activity and high anti-biofilm potential, as well as their very low host toxicity.^22^

Different types of nanoparticles such, as metal oxides, and polymeric ones are effective in reducing microbial binding, proliferation, and biofilm growth, among these nanoparticles, metal nanoparticles show effective results.^23^ The interaction of nanoparticles with microorganisms begins with the connection of these nanoparticles with their cell walls and membranes.^24^ The antimicrobial properties of silver are well known. Also, nano-silver coatings are used for medical implants such as catheters, heart valves, and wound dressings.^23*b*,25^ Silver nanoparticles have a unique mechanism for killing bacterial cells. They attach to the cell wall, causing the membrane rupture, and the accumulation of peroxides causes the cell wall to oxidize. Then the bacterial respiratory chain is attacked, thereby reactive oxygen species and hydroxyl radicals destroy the bacterium.^26^

Silver nanoparticles are biocompatible because mammalian cells can phagocyte and degrade them with lysosomal function, therefore toxicity and damage caused by free radicals are reduced and eliminated.^27^ Organic and inorganic nanoparticles can be modified by adding molecules (hybrid nanoparticles) and thus increasing their biological properties and making them multifunctional. One of them that can be attached to nanoparticles is aptamer.^28^ Aptamers are short RNA or single-stranded DNA oligonucleotides (usually containing 20 to 80 nucleotides with a molecular weight of 6 to 30 kDa) that can be folded into unique three-dimensional conformations.^29^

Aptamers have excellent selectivity and sensitivity for various molecules as targets, such as proteins, nucleotides, peptides, antibiotics, small molecules, and cells. Small size, simple handling, fast and cost-effective production, low immunogenicity, and high flexibility are the characteristics of aptamers.^30^ Some bioinformatics software such as Resetta, FR3D, and MPBind are used to predict the three-dimensional structure of aptamer.^31^ Compared to the free aptamer, the aptamer–nanoparticle complex is more resistant to nucleases. One of the outstanding features of aptamers attached to nanoparticles is their multivalent binding ability, making them more active than free aptamers.^32^ Among four DNA bases (A, T, C, and G) C and G provide excellent binding sites for Ag^+^.^33^ The nanoparticle–ligand complex exhibits many unusual properties, such as co-bonding power, high affinity, extraordinary catalytic properties for signal enhancement, and dramatic intracellular stability, making it an intracellular therapeutic and diagnostic agent.^32*b*^

In this research, by use of bioinformatics tools, a specific aptamer for *S. mutans* was designed and optimized. Due to the anti-biofilm properties of silver nanoparticles, this nanoparticle was synthesized in a new method and then combined with the designed aptamer. This complex, for the first time, was applied on streptococcal biofilm on the surface of a medical-grade titanium substrate. This research could provide the ground for preliminary studies to develop a new method for the treatment of infections related to biofilm.

## 2 Materials and methods

### 2.1 Data collection

Information regarding the sequence of fibronectin/fibrinogen-binding protein (FBP) (ARS62725.1) from *S. mutans* was collected from the NCBI (https://www.ncbi.nlm.nih.gov/search/all/?term=ARS62725.1%20). Since there was no suitable crystallography structure for this protein, 3-D modeling of this protein was performed using Galaxy web protein modeler (https://galaxy.seoklab.org/cgi-bin/submit.cgi?type=DOM). The results of crystallography were confirmed using Ramachandran plot assays.

In the case of DNA aptamer selection and optimization, the initial structure named SMa#G10-11 was used, which was previously reported by Savory *et al.* They used this aptamer (CAACCTGCTTATCTAAGGGGGGGAGGGGGGGTTGTGGGTAGGT) for specific detection of *S. mutans*.^34^

### 2.2 *In silico* selection, optimization, properties, and synthesis of aptamer for streptococcal FBP

Streptococcal surface proteins were analyzed and among different proteins, FBP with a molecular weight of 130 kDa was selected as the target.^35^ FBP sequences of different strains of *viridans streptococci* including *S*. *mutans*, *S*. *mitis*, *S*. *infantis*, and *S*. *gordonii* were obtained from the NCBI Web site, (https://www.ncbi.nlm.nih.gov/). Then, to check the similarity of these sequences, the Multalin web server, (http://multalin.toulouse.inra.fr/multalin/)^36^ was used to align the sequences, which showed a high degree of similarity between sequences. Among these bacteria, *S. mutans* was selected for further studies.

Because the structure of FBP in this bacterium was not fully available in the relevant articles or websites, including the protein data bank (PDB), by using Galaxy web online server (https://galaxy.seoklab.org/cgi-bin/submit.cgi?type=DOM)^37^ the third structure and PDB file related to the target protein were obtained. Verification of FBP modeling was performed using the Ramachandran plot from ProFunc in PDBsum to have a complete set of analyses, including PROCHECK plots to identify the likely biochemical function of a protein from its 3-D structure, (https://www.ebi.ac.uk/thornton-srv/databases/cgibin/pdbsum/GetPage.pl?pdbcode=index.html).^38^

The quality of the model was verified using Qmean, (https://swissmodel.expasy.org/qmean/)^39^ and ProSA tools, (https://prosa.services.came.sbg.ac.at/prosa.php).^40^ In the next step, the glycosylation status of the protein was investigated using of GLCOPP v1.0 web server, (https://webs.iiitd.edu.in/raghava/glycopp/). This is a web server for glycosylation sites prediction in prokaryotes.^41^

From the reported aptamers, 13 sequences were selected (Table 1). The linear secondary structure was predicted using the UNAFold online server, (https://www.unafold.org/mfold/applications/rna-folding-form.php).^42^ The folding temperature was adjusted to 37 °C in ionic conditions containing 1 M NaCl and aptamers with the minimum Δ*G* energy were selected. In the UNAFold server png output is the 2-D structure of the aptamer and dot-bracket formats (Vienna output) were used for the construction of the aptamer 3-D structures. The output was entered into the RNAComposer web server (https://rnacomposer.cs.put.poznan.pl/)^43^ to obtain the corresponding 3-D structure as a PDB file. RNA Composer output is a 3-D structure of RNA form of aptamers in which an additional hydroxyl (OH) group is present in a 2′-carbon atom of ribose and thymine is replaced by uracil. BIOVIA Discovery Studio Visualizer software (windows version v19.1.0.18287) was used to modify the aptamer 3-D structure from RNA to ssDNA. After that, the final and modified file of each aptamer was obtained.

**Table 1:** Selected aptamers for *S. mutans*

Aptamer	Sequence	Ref.
SMa#G11-6	ATACTATCGCATTCCTTCCGAGGGGGGAGGGGGGGGTG	34
	GGGGTCGGT	
SMa#G11-5	ATACATCTTAAGTCTCGTGGGGGGAGGGGGGGTTGGTG	34
	GGCTTT	
SMa#G11-20	CTACGTCTAGATTCCAGTCGAGGGGGGAGGGGGGGTTT	34
	TGGATCGGT	
SMa#G10-6	ACACCAGCGTATTCTCTTGGGGGGAGGGGGGGTTGGG	34
	GGTCGGT	
SMa#G10-11	CAACCTGCTTATCTAAGGGGGGGAGGGGGGGTTGTGG	34
	GTAGGT	
ASM-1	GCATCGGTCCTGAAGTTGCTCTAGTGCCCGTGTGCTCAA	44
ASM-2	GGGCTAGCCCCGGATCACCACTTTCCCTGCTTGATGCAA	44
ASM-3	TGTAACGGTGGAGTCGTGTTGAGGAGGCGCAATGCGTAA	44
ASM-4	TGGCACCGTGGAGTCGTGTTGAGGAGGCGCAATGCGTAA	44
ASM-5	CCTAACGTTCTCTCCTCGCTCCTCAAGGAGCCACGCTAA	44
ASM-6	CGGTTTTCGCGCTATTTCCGTACAACCCGCGACGCCTAA	44
ASM-7	GTGTTTCGCGCTATTTCCGTACAACCCGCGGACGCCTAA	44
FAM	GCAATGGTACGGTACTTCCCAAAAGTGCACGCTACTTTGCTAA	45

To select the best aptamer, docking between FBP as a receptor and aptamer as a ligand was performed by H-DOCK online web server, (https://hdock.phys.hust.edu.cn/). H-DOCK performs protein–protein and protein-DNA/RNA docking based on a hybrid algorithm of template-based modeling and *ab initio* free docking.^46^ Between these aptamers, SMa#G10-11 with 60% guanine/cytosine content, free energy (Δ*G*) equivalent to −3.16 (kcal mol^−1^), and docking score of −423.04 was used as the best-selected aptamer for further studies and optimization processes. For this purpose, the target sequence was affected by various mutations such as deletion, addition, and replacement, and a library containing 3418801 sequences was generated. By subsequent studies on the secondary structure of these sequences by the UNAFold server and examining the interactions between aptamers and the FBP sequence, the desired sequence was isolated from the other sequences.^47^

The binding sites of aptamer and FBP were studied using a full automated protein-ligand interaction profiler (PLIP) online web server, (https://plip-tool.biotec.tu-dresden.de/plip-web/plip/index).^48^ PLIP is used for identifying non-covalent interactions between biological macromolecules and their ligands.

From all the studied structures, a desirable sequence (AptBH) with the highest dock score compared to SMa#G10-11 was selected as the best aptamer. AptBH aptamer was ordered and purchased from Generay Biotech (Shanghai) Co., Ltd.

### 2.3 Synthesis of silver nanoparticles

In this study, the synthesis of silver nanoparticles (AgNPs) was carried out by a new approach of thermal decomposition. This new and simplified method was used by Tariqul Islam *et al.* for the synthesis of zinc oxide nanoparticles.^49^ For this reason, silver nitrate (AgNO_3_) was obtained from Sigma-Aldrich, and sucrose was purchased from AppliChem GmbH. Before synthesizing AgNPs, an electric furnace was preheated to about 500 °C. In a 250 mL Pyrex glass beaker, 1076 mg of sucrose and 3202 mg of AgNO_3_ salt were mixed with 3 mL of deionized water (DW) and the beaker was placed in the electric furnace for 40 min. After 40 min of heating, the AgNPs powder was allowed to cool down. For cooling, normal ambient cooling was utilized. The silver-color powder was harvested with a spatula. The amount of AgNPs obtained was about 330 mg, which was stored under ambient conditions. The obtained powder was characterized using scanning electron microscopy (SEM, Philips XL30), powder X-ray diffraction (XRD, D8 ADVANCE, Bruker), and dynamic light scattering (DLS, SZ100, Horiba) methods.^50^

### 2.4 Conjugation of silver nanoparticles with DNA aptamers

For this step, at first, AgNPs were functionalized with streptavidin (SA), then conjugated with AptBH aptamer. The reported method by Li Qiang *et al.* was used to perform this section. Briefly, 80 μL of 1 mg mL^−1^ SA was added to the AgNPs suspension (2 mL, 2.43 nM) at pH 6.7 (adjusted with 0.1 M K_2_CO_3_), and incubated using a rotary shaker at 37 ± 0.2 °C for 120 min.

The mixture was then centrifuged at 10 000 rpm for 15 min. After that, the sediment was washed twice and resuspended in PBS solution to gain SA-AgNPs. Biotin-aptamer in PBS buffer (10 μL, 33.33 nM) was added to the SA-AgNPs and incubated at 37 ± 0.2 °C for 15 min. The final product (AgNPs–AptBH) was acquired by centrifugation at 9000 rpm for 15 min. The pellet was resuspended in PBS containing 0.1% BSA. The hydrodynamic diameter of nanoparticles suspension was measured with DLS.^51^ Gel retardation assay (1% agarose gel electrophoresis) was performed for DNA ladder, AptBH, AgNPs, and AgNPs–AptBH.^52^

### 2.5 Biofilm formation and suppression assay

For biofilm formation, *S. mutans* (PTCC1683) was obtained from the Iranian Research Organization for Science and Technology (IROST). Brain heart infusion (BHI) containing sucrose (supplementary agent at 2%) was used as a medium for *S. mutans* biofilm formation. For quantitative and qualitative inhibition of biofilm formation and destruction formed biofilm study on the surface of medical-grade titanium, the microtiter plate (MTP) method by crystal violet (CV) was used.^53^ In each well of a 96-flat bottom microtiter plate, a small piece of titanium was placed, and 200 μL of BHI containing 2% sucrose was distributed and inoculated with 20 μL of a fresh overnight culture of *S. mutans* and then incubated at 37 ± 0.2 °C for 48 h.

To investigate the inhibition effect of free AgNPs and AgNPs–AptBH on biofilm formation, before incubation, the concentrations of 12.5, 25, 50, 75, and 100 μg mL^−1^ of free AgNPs and the same amount of AgNPs–AptBH were added to the 96 microtiter plate. After this step, the plate was incubated at 37 ± 0.2 °C for 48 h. Then, the liquid culture was removed by inverting the plate and decanting the liquid with a gentle flick of the wrist. The plate was then immersed in a large beaker containing deionized water (DW). After that, the DW in each well was removed. This process was repeated two times, incubating washed plate at 60 °C for drying and fixation of adherent cells before staining. Staining was performed with a 1% (w/v) solution of CV dissolved in DW. The amount of CV bound in each well is proportional to the amount of biofilm. To stain biofilms, 50 μL 1% CV was dissolved in DW in each well and allowed at least five minutes for staining.

After the staining reaction had been completed, the excess stain was removed by repeated washing (3–4 washes) with DW. The wash solution should be clear after the final last washing step. The CV can be eluted from stained biofilms by adding 200 μL ethanol 96% to each well, titanium pieces were removed and the eluted dye was transferred to a fresh assay plate, and OD_630_ was measured using an appropriate microplate reader (Bio Tek, 800 TS microplate reader, USA).^54^ The percentage of inhibition was calculated using eqn (1):^55^

1 Inhibition (%) = [(OD_control_ − OD_treatement_)/OD_control_] × 100

To investigate the degradation effect of AgNPs–AptBH, and free AgNPs, the same method as above was done, but biofilm formed on titanium for 48 h. After that, concentrations mentioned above were applied in the 96 well flat bottom microtiter plate after biofilm formation for 24, and 48 h, in the same manner as the above coloring was done.

The percentage of biofilm degradation was calculated using the following equation:^56^

2 Biofilm degradation (%) = 1 − [(OD_treatement_)/OD_control_] × 100

For qualitative investigation of anti-biofilm effects, biofilm was formed on titanium for 2 weeks, and then 100 μg mL^−1^ of AgNPs, and the same amount of AgNPs–AptBH was applied to these biofilms at the time 24, and 48 h after biofilm formation. After that, biofilm was fixed on titanium by sinking it on glutaraldehyde 2.5% for 4 h, then sinking titanium in ethanol 10, 20, 30, 40, 50, 60, 70, 80, 90, and 100% for 15 min in each step. Afterward, these samples were investigated by SEM and the results were compared.^57^ In this study, all experiments were performed thrice, and multiple comparisons in 2-way ANOVA were used for statistical analysis.

### 2.6 Stability and cell viability assay

The stability of AgNPs–AptBH was investigated by the decomposition of this complex at 450 nm in a cell culture medium for 360 min. For the biocompatibility (cell viability) test, the MTT assay was used. The MTT assay is a test to check for cell viability or the toxicity of drugs or other supplements on the cell, differentiating between living and dead cells by affecting intracellular organs.^58^ MTT assay, which stands for MTT 3-(4,5-dimethylthiazol-2-yl)-2,5-diphenyltetrazolium bromide, is a colorimetric method.^59^ For this purpose, MTT was done on the MCF7 (breast cancer cell line) growing cells in the logarithmic phase. The advantage of the MCF-7 cell line is that it is known as an investigative tool that led to its acceptance in laboratories around the world.^60^

The cells were first seeded in a 96-well culture plate (7 × 10^3^ cells per well), and after 24 h of incubation and ensuring that the cells were alive and sticking to the floor wells, treatment of cells with different concentrations of AgNPs, and AgNPs–AptBH (12.5, 25, 50, 75, and 100 μg mL^−1^) were performed. The plates were incubated at 37 ± 0.2 °C with 5% CO_2_ for 24 h. After that, the contents of the plate were drained under sterile conditions, and 20 μL of 5% MTT solution was added to each well, and the plate was incubated again for 4 h under the same conditions as above. After this time, the contents of the MTT were drained and 100 μL of DMSO was added to each well, and finally, the absorbance was measured at 630 nm.^61^

The percentage of cell viability was calculated using eqn (3):^62^

3 Cell viability (%) = (OD_Sample_/OD_Control_) × 100

### 2.7 Hemolytic effect of free AgNPs, and AgNPs–AptBH on red blood cells

To investigate the hemolytic effect of AgNPs, and AgNPs–AptBH complex, first, 5 mL of human fresh blood was diluted with 10 mL of PBS and then centrifuged at 1500 rpm for 15 min. The supernatant was discarded and the red blood cell pellets were washed 2-times with 10 mL of PBS. Then, the resulting precipitate was suspended in 50 mL of PBS. Red blood cells in DW suspension were used as positive control and buffer suspension (contained RBC) was used as a negative control. After this step, from each concentration 12.5, 25, 50, 75, and 100 μg mL^−1^ of free AgNPs and AgNPs- AptBH, were added to one milliliter of erythrocyte suspension in the microtubes. Then these microtubes were incubated at 37 ± 0.2 °C for 3 h in a shaker incubator, and after this time, the microtubes were centrifuged again at 1500 rpm for 5 min. 200 μL of each supernatant was transferred to a 96-well plate, and the sample's OD was read at 630 nm.^63^ Each test was repeated three times. The hemolysis percentage was calculated using eqn (4):^64^

4 Hemolysis (%) = [(OD_sample_ − OD_negative control_)/(OD_positivecontrol_ − OD_negativecontrol_)] × 100

## 3 Results and discussion

### 3.1 *In silico* selection of effective aptamers against streptococcal FBP

The FBP from *S. mutans* protein (ARS62725) has two main conserved domains: N-terminal domain as binding domain (FbpA) and unknown functional domain as DUF814 (https://www.ncbi.nlm.nih.gov/protein/ARS62725.1?report=graph).

By entering FASTA-formatted protein sequences of FBP to the GLCOPP v1.0 online web server and choosing prediction based on Binary Profile of Patterns (BPP), it was found that this protein has no potential for O-linked and N-linked glycosylation sites. Therefore, FBP has no steric effect.

**Fig. 1:**
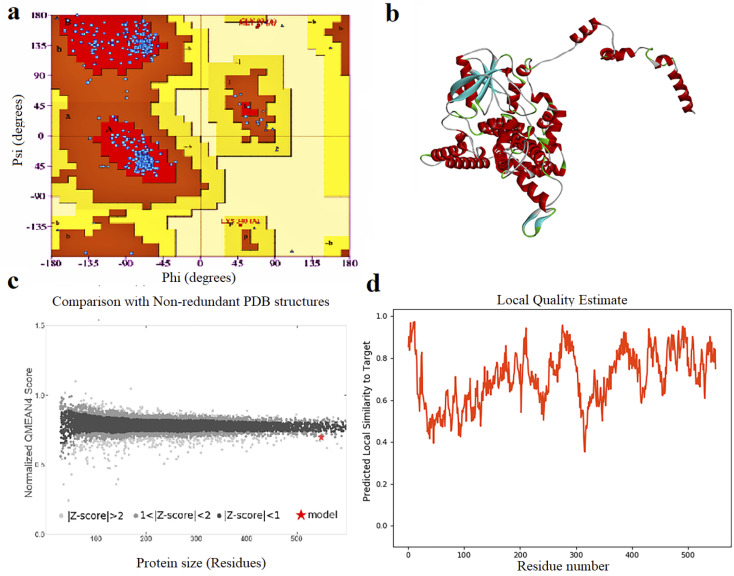
(a) Ramachandran plot analysis graph, (b) 3D structure, (c) quality comparison and (d) local quality estimate of FBP construct.

The results of 3-D modeling prediction were confirmed using Ramachandran plot assays. Ramachandran plot statistics for FBP showed that 94.1% of residues were in the most favored regions (A, B, L, which contain 475 residues), 5.3% of residues (27 residues) were in allowed regions (a, b, l,p), 0.2% of residues (1 residue) was in generously allowed regions (∼a, ∼b, ∼l, ∼p) and 0.4% of residues were in disallowed regions (XX). Based on an analysis of 118 structures of resolution of at least 2.0 angstroms and R-factor no greater than 20.0, a good quality model would be expected to have over 90% in the most favored regions (Fig. 1).

In the aptamer selection and optimization process for FBP, at first, for 13 collected sequences some properties such as GC content percentage, Δ*G* (kcal mol^−1^), and docking score (H-DOOCK score) were calculated (Table S1†). Among these aptamers, SMa#G10-11 had a high docking score (−423.04), the lowest free energy among the complexes (Δ*G*_aptamer_ = −3.16 kcal mol^−1^), and a GC content of 60%.

The sequence of the original is CAACCTGCTTATCTAAGGGGGGGAGGGGGGGTTGTGG GTAGGT, which was introduced by Savory *et al.* (2014) against *S. mutans* (33). After optimizing the original aptamer, the new aptamer (AptBH) with a sequence of 5′AAACCTGCTTATCTAAGGGGGGGAGGGGGGGTAGTGGGTGGGT-biotin-3′ was selected for this research. It has a GC content of 60%, a molecular weight of 13581.6 kDa, and a melting point of 77.92 °C.

**Fig. 2:**
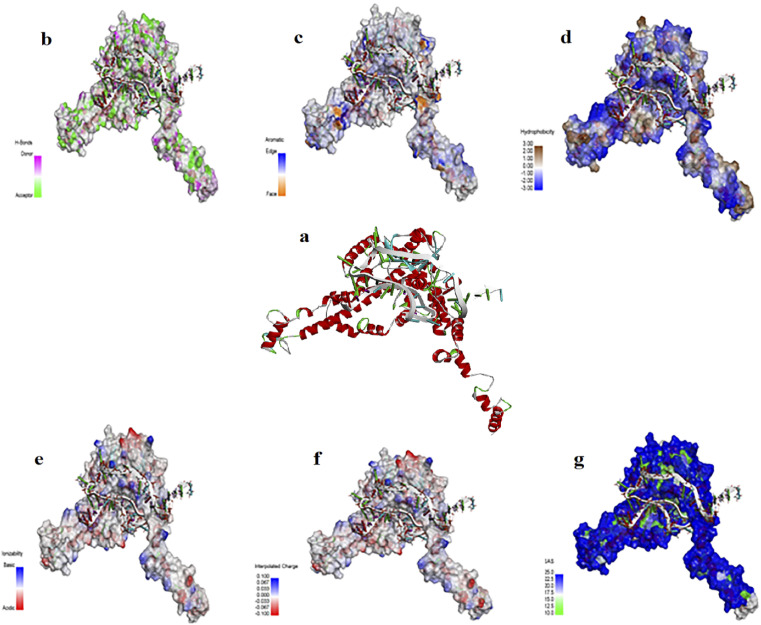
(a) The interaction of Aptamer AptBH with the FBP. FBP surface interactions with AptBH with respect to surfaces of (b) hydrogen bonds, (c) aromatic interactions, (d) hydrophobicity, (e) ionizability, (f) interpolated charges, and (g) solvent accessibility.

2-D and 3-D structures of this aptamer are shown in Fig. S1.† The docking results of the aptamer (*i.e.*, AptBH) with the receptor (in this study = FBP) and the levels of hydrophobic interactions, hydrogen bonds, π-stacking, and salt bridges are shown in Fig. 2. Also, the interaction sites for the aptamer and FBP of *S. mutant* were illustrated by the PLIP web server (Table S2†).

### 3.2 Synthesis of silver nanoparticles

**Fig. 3:**
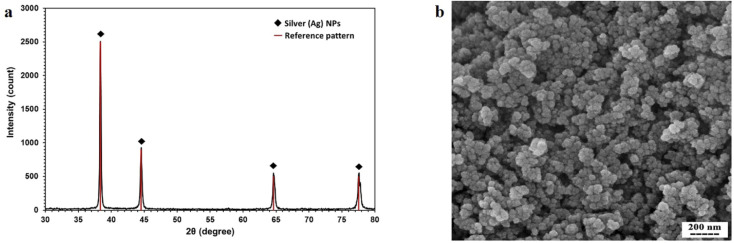
(a) XRD pattern of AgNPs synthesized by thermal decomposition method. (b) SEM micrograph of synthesized AgNPs.

The crystal structure of the synthesized AgNPs was identified by the XRD method. As shown in Fig. 3a, the corresponding XRD pattern of the synthesized nanoparticles in this work confirms that the powder is silver.

The result of XRD was analyzed by X'Pert HighScore Plus software. According to Fig. 3a, this pattern has 4 peaks by intensity 999, 451, 223, and 221 which correspond to 38.26, 44.47, 64.71, and 77.74 2θ angles respectively with reference code pattern 01-087-0719, indicating and proving the presence of silver nanoparticles. Due to the absence of other unrelated peaks, the sample synthesized in this study contains only AgNPs.

In the next step of synthetic nanoparticle characterization, DLS was done for finding the hydrodynamic radius of AgNPs. The results of the DLS test show that the size of nanoparticles varies from 50 nm to 70 nm.

The SEM image showing the morphology of nanoparticles is presented in Fig. 3b. The nanoparticles were relatively spherical, and the single nanoparticles that were glued together showed larger nanoparticles.

### 3.3 Selective aptamer conjugation to silver nanoparticles

The hydrodynamic diameter of AgNPs and AgNPs–AptBH were obtained in ranges of 50–70, and 78–99 nm in size, respectively. The hydrodynamic diameter of binary mixtures of AgNPs–AptBH compared to AgNPs showed an increase in hydrodynamic diameter that confirmed the successful attachment of aptamers to the nanoparticles.

Gel retardation assay was done to compare the aptamer, Ag NPs, and AgNPs–AptBH motility. The result showed that the rate of aptamer migration is significantly rather than AgNPs and AgNPs–AptBH. The brightness of the bands on the gel showed conformational stability of AgNPs–AptBH (Fig. S2†).

### 3.4 Anti-biofilm assay

**Fig. 4:**
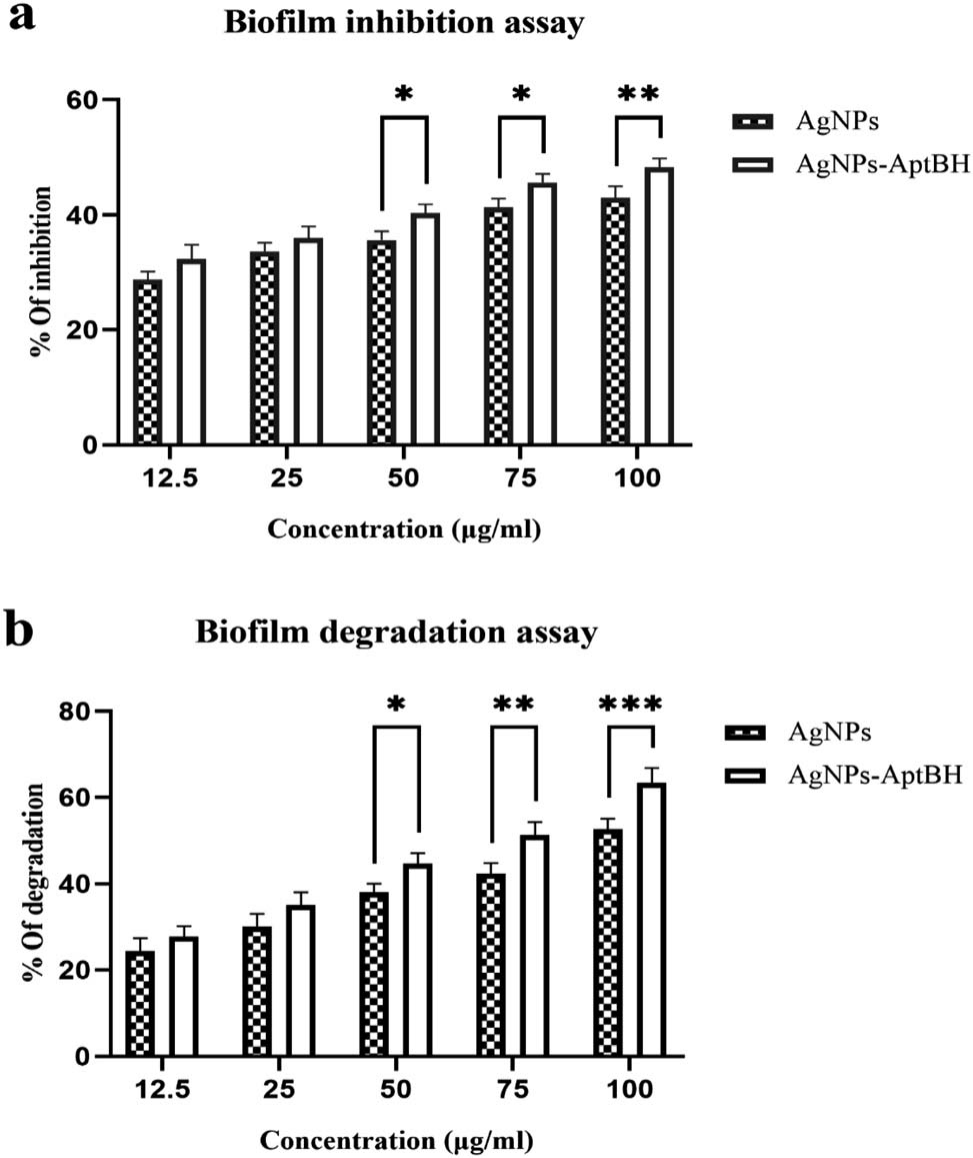
(a) Biofilm inhibition assay and (b) degradation assay for 48 h in presence of AgNPs and AgNPs–AptBH. (in (a), *p* value = 0.6429 non-significant difference, ****p value < 0.0001 significantly different, and in (b), *p* value = 0.1953 non-significant difference, ****p value < 0.0001 significantly different). Error bars were estimated from three replicate samples.

The effects of free AgNPs, and AgNPs–AptBH could inhibit biofilm formation in a dose-dependent manner. However, the anti-biofilm activity at the low dose of nanoparticles (12.5, and 25 μg mL^−1^) was not significantly different. However, the anti-biofilm activity increased by increasing the concentration from 25 to 100 μg mL^−1^ (Fig. 4a).

Biofilm degradation at the low doses (12.5, and 25 μg mL^−1^) of nanoparticles and complex (AgNPs–AptBH) have a similar degrading effect. There is no significant difference between them, but with increasing the dose (50, 75, and 100 μg mL^−1^), the destructive effect of nanoparticles and complex differs significantly (Fig. 4b). Aptamer effectively and purposefully binds nanoparticles to biofilm and enhances its effectiveness.

**Fig. 5:**
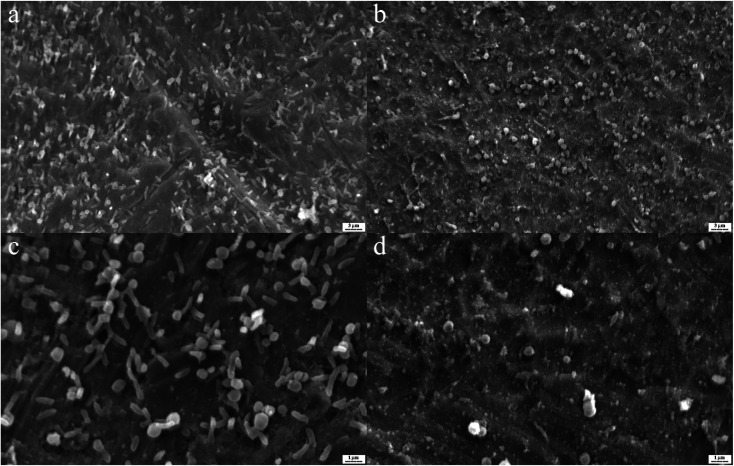
*S. mutans* biofilms on the titanium as base matrix treated with 100 μg mL^−1^ AgNPs–AptBH (a and c), and 100 μg mL^−1^ AgNPs (b and d).

In Fig. 5, by use of SEM imaging, the qualitative effect of silver nanoparticles and their conjugate to aptamer could be seen. By comparing these SEM pictures difference between the effect of silver nanoparticles and complex on biofilm can be understood. The AgNPs–AptBH has a more significant effect rather than free AgNPs on the formed biofilms.

### 3.5 Stability and cell viability analysis

**Fig. 6:**
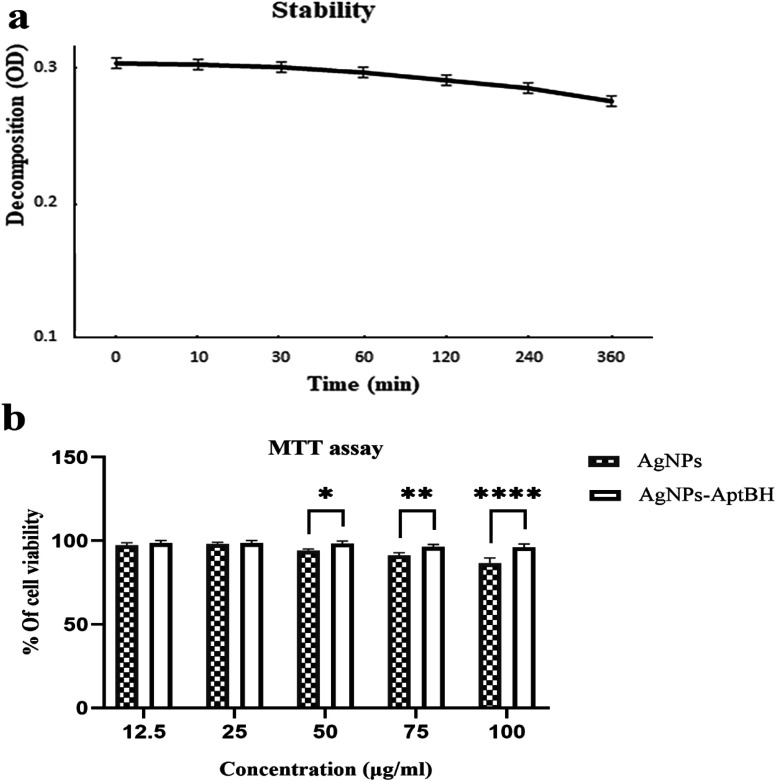
(a) Stability of the AgNPs- AptBH in cell culture medium. (b) MTT assay shows minor toxicity to Mcf7 cells in presence of AgNPs–AptBH rather than AgNPs. Error bars were estimated from three replicate samples. (*p* value > 0.8855 non-significant difference, ****p value < 0.0001 significantly different).

Fig. 6a shows that the AgNPs–AptBH complex has certain stability over 360 min and has not decomposed overtime for 360 min and is stable in the cell culture medium. The MTT assay revealed that the toxicity and therefore biocompatibility of AgNPs, and AgNPs–AptBH to cells was mainly dependent on the dose of nanoparticles in the cell medium (Fig. 6b). When the concentration of AgNPs–AptBH was 100 μg mL^−1^, cell availability could reach 96%, suggesting that this dose of AgNPs–AptBH is weakly toxic. In compare of it, in the case of applying the same dose of free AgNPs, cell viability could reach 87%, so it can be said that AgNps-AptBH has less toxicity rather than free AgNPs. In the lower doses (12.5, and 25 μg mL^−1^) there are no significant differences but by increasing the dose to 50, 70, and 100 μg mL^−1^, the toxicity effect goes up. So that AgNPs–AptBH rather than AgNPs is more biocompatible.

### 3.6 Hemolytic effect of free AgNPs and AgNPs–AptBH on red blood cell

**Fig. 7:**
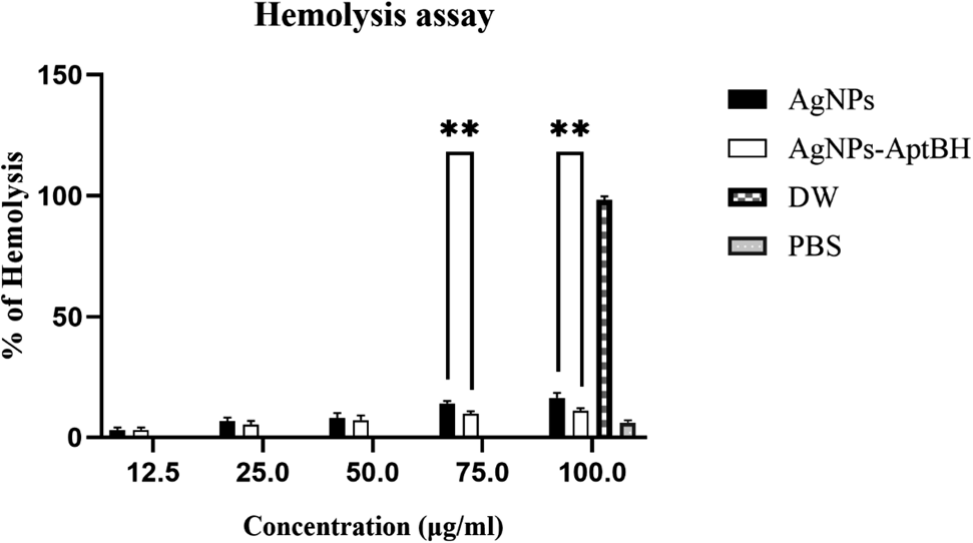
Percentage of hemolysis of RBCs incubated with AgNPs and AgNPs–AptBH at different concentrations, ranging from 12.5 to 100 μg mL^−1^ for 3 h. (*p*-value >0.8164 non-significant difference, ****p value < 0.0001 significantly different).

Fig. 7 and S3† shows the hemolytic effects of these two groups were dose-dependent. At the lower doses (12.5, 25, and 50 μg mL^−1^) there is no significant difference between AgNPs and AgNPs–AptBH, but at the upper doses (75, and 100 μg mL^−1^) significant differences could be seen. AgNPs and AgNPs AptBH at the concentration of 75 μg mL^−1^ caused 14, and ∼9% hemolysis and at the 100 μg mL^−1^ caused ∼16, and 11% respectively. The only difference between these two groups is the binding of the aptamer to one and the absence of aptamer in the other, therefore it can be said that the binding of the aptamer to silver nanoparticles has increased its biocompatibility to some extent.

Today, *in silico* study by use of bioinformatics tools including databases, online and offline servers, and existing software has become one of the fields of interest for designing and optimizing aptamers.^31,65^ In this research, a specific aptamer for *S. mutans* was designed and optimized using *in silico* methods. The designed aptamer was attached to silver nanoparticles and showed potent anti-biofilm activity against streptococcal biofilm.

Jaiswal *et al.* (2015) reported the silver nanoparticles stabilized on beta-cyclodextrin, have excellent anti-biofilm properties, and can prevent the formation of *Staphylococcus epidermidis* biofilm, which is a common microorganism in synthetic implant and catheter biofilms. They could inhibit up to 90% of the biofilms using different concentrations of silver nanoparticles.^66^ Several reports demonstrated the anti-biofilm effects of silver nanoparticles against a range of clinical microorganisms under static conditions in a bioreactor. Martinez-Gutierrez *et al.* (2013) investigated the antimicrobial effects of silver nanoparticles on biofilms obtained under static conditions and biofilms obtained under liquid currents in a bioreactor. Their results showed that these nanoparticles could effectively inhibit biofilm formation and kill bacteria in the biofilm.^67^

Some researchers have declared that silver nanoparticles, alone and combined with conventional antibiotics, have therapeutic potential to inhibit and degrade biofilm infections. Gurunathan *et al.* (2014) were biologically produced silver nanoparticles and reported that the combination of these nanoparticles with ampicillin and vancomycin showed a synergistic effect on biofilm formation against Gram-negative and Gram-positive bacteria, respectively.^68^

In some studies, researchers used silver nanoparticles to reduce the protein and carbohydrate content of the biofilm matrix. Namasivayam *et al.* (2013) in their study showed that silver nanoparticles could weaken the biofilm and deliver a drug to it.^69^ In comparison with these studies, the free nanoparticles synthesized in our study were able to prevent up to 43% of biofilm formation and eradicated the formed biofilm by *S. mutans* up to 53%.

In the recent decade, some studies used aptamers in molecular targeting and reported many potential advantages over traditional antibodies. In 2017, Lijuan *et al.* designed an aptamer and connected it to ampicillin. This aptamer facilitated ampicillin entrance into the biofilm and reduced its antibiotic load tolerance.^70^ Wang *et al.* (2017) designed an aptamer, which acted as a delivery agent to the target. Their complex (aptamer–ciprofloxacin–SWNT) also had very high anti-biofilm activity compared to its components.^71^ In 2018, Mao *et al.* used graphene oxide and aptamer bound to graphene oxide to target *Salmonella typhimurium* biofilm. Aptamer attached to graphene oxide prevented the formation of biofilm up to 93.5% and destroyed up to 84.6% of the relevant biofilm.^72^

Some researchers have used aptamer conjugated to nanoparticles for human immunodeficiency virus (HIV) therapeutics purposes. Shiang *et al.* (2013) designed an aptamer for HIV-1 and attached it to the gold nanoparticles. By use of this complex, they dramatically inhibited the reverse transcriptase enzyme involved in the replication of the virus.^32*b*^ In some reports by conjugation of aptamers to specific parts of viruses, researchers removed viruses from samples' blood. Delaviz *et al.* (2015) designed an aptamer that specifically binds to the glycoprotein of the virus to remove hepatitis C virus particles from the plasma and bind it to iron oxide magnetic nanoparticles. By use of the aptamer–magnetic nanoparticle complex, the researchers removed large amounts of viral particles from the plasma samples.^73^

In 2014, Zamay *et al.* designed a DNA aptamer that binds specifically to vimentin. This aptamer causes the programmed death of cancer cells in mice. They used natural arabinogalactan polysaccharide as a carrier to deliver aptamer. During five days of injection of aptamer–arabinogalactan complex, growth of adenocarcinoma was effectively inhibited.^74^ In our study, the synthesized silver nanoparticles were conjugated to a specific aptamer for *S. mutans*. This complex was able to prevent biofilm formation up to 53% and eradicate up to 63% of the formed biofilm.

## 4 Conclusion

The binding of nanoparticles to biomolecules has become an attractive part of research related to biocompatible drug nanoparticles, effective drug delivery machines, biosensors, bio-analyzers, and the next generation of antibiotics. This study indicated that the aptamer conjugated to silver nanoparticles could effectively reduce the formation of biofilm and even destroy the formed biofilm *in vitro*. The efficient anti-biofilm activity of this nano-complex, along with having no hemolysis and toxicity properties make this complex a potential therapeutics against the disorders related to *S. mutans* biofilm. Future more studies are needed to investigate, develop and improve the effectiveness of this complex *in vivo* so this can be helpful to confirm the application of the conjugated nano-system *in vivo*.

## Conflicts of interest

The authors declare that they have no conflicts of interest.

## Supplementary Material

RA-012-D2RA04112J-s001
