# Supplementary material for: Investigating the anti-streptococcal biofilm effect of ssDNA aptamer-silver nanoparticles complex on a titanium-based substrate
Source: RSC Adv. 2022 Sep 15;12(38):24876–86. doi: 10.1039/d2ra04112j (PMC9475424; doi:10.1039/d2ra04112j)
Supplement: RA-012-D2RA04112J-s001 [file RA-012-D2RA04112J-s001.pdf]

Electronic Supplementary Material (ESI) for RSC Advances.
This journal is © The Royal Society of Chemistry 2022

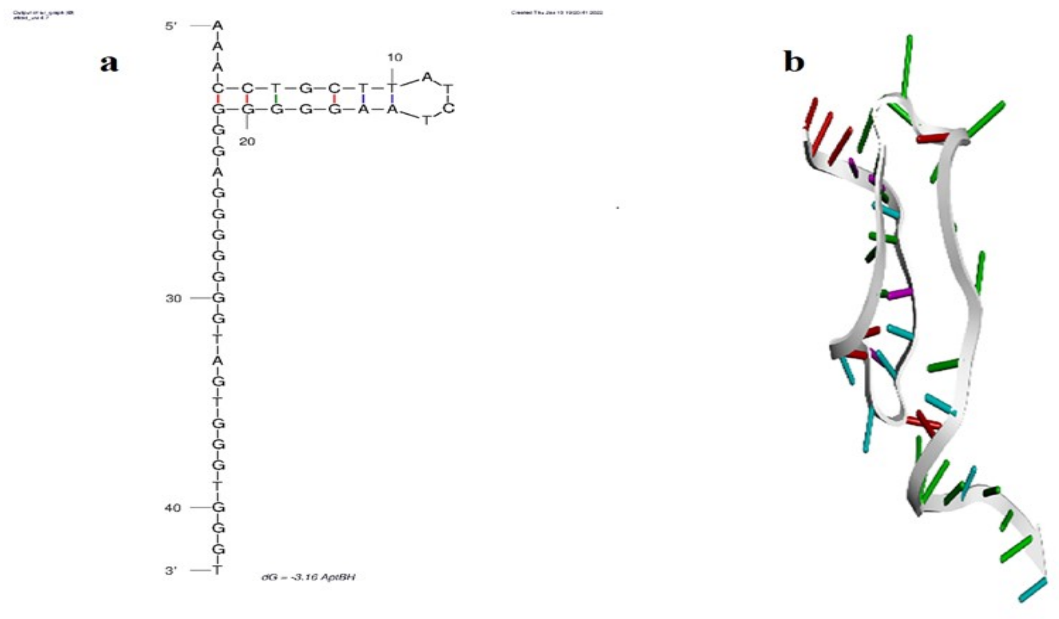

Figure s1. a) 2-D and, **b)** 3-D structures of AptBH.

Table s1. Free energy content, guanine/cytosine percentage of selected aptamers, and corresponding docking score.

**Aptamer**	**GC%**	**$\Delta G_{aptamer}$(kcal/mol)**	**Docking Score**
**SMa#G11-6**	66	-3.16	-313.86
**SMa#G11-5**	57	-0.20	-300.01
**SMa#G11-20**	60	-0.53	-332.89
**SMa#G10-6**	66	-1.47	-271.23
**SMa#G10-11**	60	-3.16	-423.04
**ASM-1**	56	-1.52	-292.26
**ASM-2**	59	-3.06	-297.61
**ASM-3**	54	-3.67	-301.65
**ASM-4**	59	-2.14	-323.57
**ASM-5**	56	-4.81	-322.15
**ASM-6**	56	-4.47	-280.24
**ASM-7**	56	-3.62	-325.28
**FAM**	47	-3.66	-287.02

Table s2. Detail of interactions between AptBH aptamer and FBP of *S. mutans*

Type of interaction	Amino acid residue	Nucleotide residue	Distance (A°)
Hydrophobic Interactions	Pro69	G36	3.02
	Phe148	G23	3.76
	Gln70	T35	3.99
Hydrogen bonds	Ser71	T12	3.07
	Thr74	T10	3.58
	Lys459	G31	3.88
	Gln507	G25	4.07
	Lys514	G31	3.85
	Lys515	G28	3.81
	Asn517	G7	3.96
	Arg530	C5	3.44
	Gly531	G27	2.99
	Lys533	G27	3.47
	Arg536	G25	3.51
Salt Bridges	Lys467	G25	2.55
	Glu512	G29	3.38
	Glu512	G30	4.47
π-Cation interaction	Arg80	T39	5.01

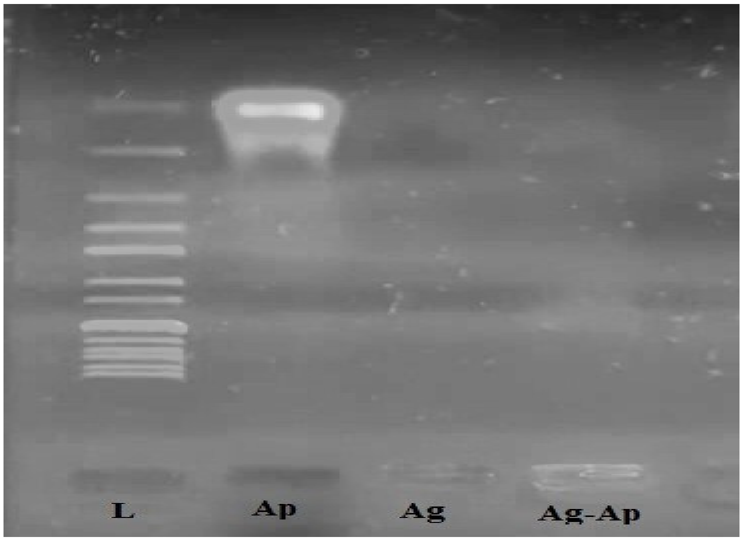

Figure s2. Gel retardation assay for DNA Ladder (L), AptBH aptamer (Ap), AgNPs (Ag) and AgNPs-AptBH (Ag-Ap)

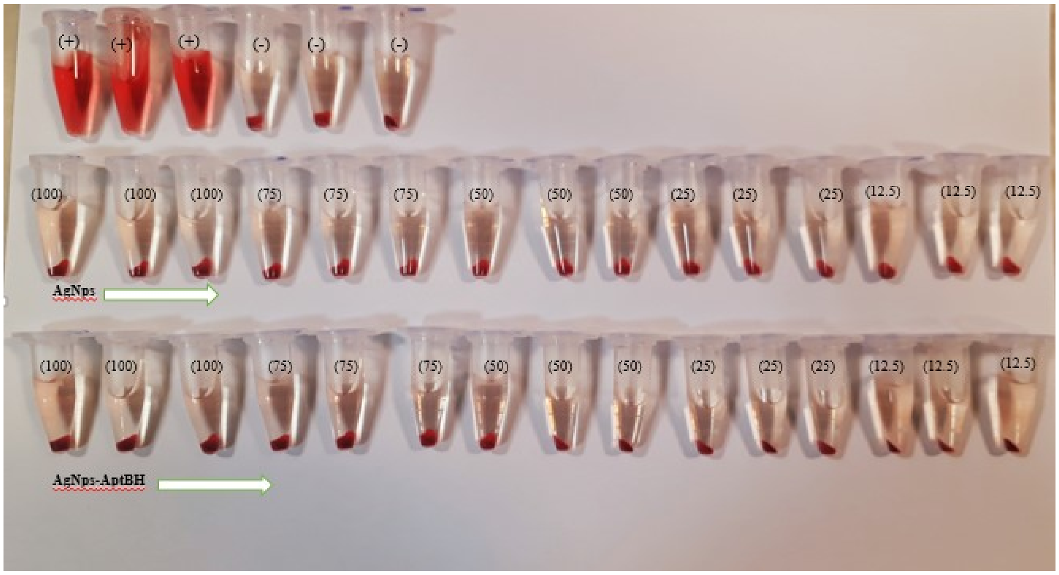

Figure s3. Photographs of hemolysis of RBCs. DW (+) and PBS (-) are used as the positive and negative control, respectively.
